# Tumor Markers and Their Diagnostic Significance in Ovarian Cancer

**DOI:** 10.3390/life13081689

**Published:** 2023-08-05

**Authors:** Alkis Matsas, Dimitrios Stefanoudakis, Theodore Troupis, Konstantinos Kontzoglou, Makarios Eleftheriades, Panagiotis Christopoulos, Theodoros Panoskaltsis, Eleni Stamoula, Dimitrios C. Iliopoulos

**Affiliations:** 1 Laboratory of Experimental Surgery and Surgical Research ‘N.S. Christeas’, Medical School, National and Kapodistrian University of Athens, 11527 Athens, Greece; 2 Second Department of Obstetrics and Gynecology, Medical School, “Aretaieion” University Hospital, National and Kapodistrian University of Athens, 11527 Athens, Greece; 3 Department of Anatomy, Faculty of Health Sciences, Medical School, National and Kapodistrian University of Athens, MikrasAsias Str. 75, 11627 Athens, Greece; 4 Department of Clinical Pharmacology, School of Medicine, Aristotle University of Thessaloniki, University Campus Aristotle University of Thessaloniki, 54124 Thessaloniki, Greece

**Keywords:** ovarian cancer, cancer biomarkers, early detection, prognosis, cancer diagnosis, CA-125, CA 15-3, CA 19-9, HE4, hCG, inhibin, AFP, LDH, precision oncology

## Abstract

Ovarian cancer (OC) is characterized by silent progression and late-stage diagnosis. It is critical to detect and accurately diagnose the disease early to improve survival rates. Tumor markers have emerged as valuable tools in the diagnosis and management of OC, offering non-invasive and cost-effective options for screening, monitoring, and prognosis. Purpose: This paper explores the diagnostic importance of various tumor markers including CA-125, CA15-3, CA 19-9, HE4,hCG, inhibin, AFP, and LDH, and their impact on disease monitoring and treatment response assessment. Methods: Article searches were performed on PubMed, Scopus, and Google Scholar. Keywords used for the searching process were “Ovarian cancer”, “Cancer biomarkers”, “Early detection”, “Cancer diagnosis”, “CA-125”,“CA 15-3”,“CA 19-9”, “HE4”,“hCG”, “inhibin”, “AFP”, “LDH”, and others. Results: HE4, when combined with CA-125, shows improved sensitivity and specificity, particularly in early-stage detection. Additionally, hCG holds promise as a prognostic marker, aiding treatment response prediction and outcome assessment. Novel markers like microRNAs, DNA methylation patterns, and circulating tumor cells offer potential for enhanced diagnostic accuracy and personalized management. Integrating these markers into a comprehensive panel may improve sensitivity and specificity in ovarian cancer diagnosis. However, careful interpretation of tumor marker results is necessary, considering factors such as age, menopausal status, and comorbidities. Further research is needed to validate and refine diagnostic algorithms, optimizing the clinical significance of tumor markers in ovarian cancer management. In conclusion, tumor markers such as CA-125, CA15-3, CA 19-9, HE4, and hCG provide valuable insights into ovarian cancer diagnosis, monitoring, and prognosis, with the potential to enhance early detection.

## 1. Introduction

### 1.1. Background of Ovarian Cancer

In the United States in 2020, there were 1,806,590 new cases of cancer and 606,520 cancer-related deaths. Specifically, OC appears as the leading cause of death for female reproductive tract cancers. It is estimated that in 2020, there were 21,750 new cases and 13,940 deaths related to OC. Postmenopausal women are considered at high risk of developing OC because the likelihood of developing an advanced stage disease increases with age. An absence of early detection methods and the limited effectiveness of standard chemotherapy are the main factors contributing to this vulnerability [1]. The prevalence of OC among post-menopausal women is estimated to be 1 in 2500 [2]. OCs are diagnosed at an advanced stage for around 70% of the cases, resulting in a 5-year survival rate of only 30%. Nevertheless, the 5-year survival rate can exceed 90% when OC is detected early and confined to the ovaries. It is essential to gain a deeper understanding of the molecular causes of OC, even though, in the last 25 years, modest improvements in survival rates have been observed. Crucially, new biomarkers could aid in the early detection pathway, especially as less than 20% of OCs are diagnosed at a localized stage [3]. 

Among gynecological malignancies, malignant epithelial tumors (carcinomas) are the deadliest forms of OC. Currently, the classification of ovarian epithelial tumors has solely relied on the morphology of tumor cells. Six to nine cases per 100,000 women is the global incidence rate of these cancers [4]. Broadly, there are three categories of OC based on the types of ovarian cells involved. Surface epithelial cells are the cell type in category one, and can cover the ovary and be subdivided into many subtypes. The second category consists of germ cells, which are the cells that ultimately develop into ova. OC subtypes related to germ cells encompass yolk sac tumors, immature teratoma, and dysgerminoma. Finally, sex cord–stromal cells comprise the third category. These tumors include malignant granulosa cells and Sertoli–Leydig cells [5,6]. 

**Figure 1:**
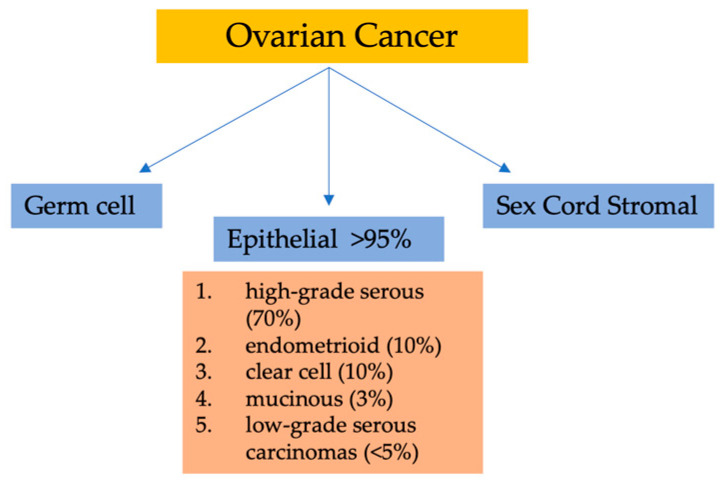
Classification of ovarian malignancies.

Considering their histopathology and molecular genetic alterations, the subtypes of epithelial ovarian cancers (EOCs) are as follows: (1) high-grade serous, (2) endometrioid, (3) clear cell, (4) mucinous, and (5) low-grade serous carcinomas. EOCs constitute more than 95% of all OC cases (Figure 1). Moreover, based on characteristics such as extent of cell proliferation, the presence of nuclear atypia, and stromal invasion, the tumors are further subdivided as benign, borderline (intermediate), and malignant (carcinoma). This detailed categorization aids in providing a comprehensive understanding of the nature and behavior of these tumors [7,8,9]. 

The dualistic model of ovarian carcinogenesis encompasses the primary histopathological subtypes, consolidating them into two distinct categories—type I and type II—based on clinical, genetic, and developmental characteristics. In terms of diagnosis, type I ovarian carcinomas constitute approximately 30% of cases, whereas type II tumors represent the majority, accounting for about 70%. Type I tumors tend to be confined to the ovaries (stage I) and generally exhibit a more favorable prognosis, contributing to a mere 10% of ovarian cancer-related fatalities. Type I tumors correspond to an initial phase characterized by clinically less aggressive behavior. These tumors typically include low-grade clear-cell, serous, mucinous, and endometrioid subtypes, with rare occurrences of seromucinous and Brenner tumors. Conversely, type II tumors display a more aggressive nature and account for the majority of cases of epithelial ovarian cancers (EOCs); they are typically diagnosed at advanced stages (III and IV), leaving limited prospects for a cure. This category comprises high-grade undifferentiated, endometrioid, serous, and malignant mixed mesodermal tumors, which are associated with poorer prognosis and clinical outcomes. Astonishingly, type II tumors are responsible for an overwhelming 90% of ovarian cancer-related deaths. Consequently, some experts advocate directing extensive screening efforts towards type II tumors as a strategic approach to potentially yield substantial improvements in patient outcomes [10,11,12,13,14].

### 1.2. Significance of Early Diagnosis in Ovarian Cancer

OC is a complicated and diverse group of diseases characterized by variations in morphology and biological behavior. Although its prevalence is less than that of breast cancer, the impact of OC is disproportionally higher with a significant number of deaths attributed to the disease. OC proves fatal for the vast majority of patients diagnosed with advanced (stage III) ovarian tumors since recurrence following surgery and chemotherapy is seen in around 75% of cases. Globally, OC is considered as the most lethal gynecological cancer and the fifth most common cause of cancer-related deaths among women in the Western world [8,9,15]. By improving the efficacy of screening methods, such as tests for specific biomarkers, the chances of detecting OC at an early stage could be increased. 

## 2. Tumor Markers in Ovarian Cancer

Biomarkers, also known as oncomarkers, play a crucial role in cancer research and treatment by providing measurable characteristics of different cell types. These molecular signatures encompass genes, proteins, and other molecular features that can serve as objective medical signs. Biomarkers serve two primary purposes: firstly, they help assess the likelihood of disease progression or pathological processes, and secondly, they aid in evaluating the response to therapeutic interventions. Cancer biomarkers are molecules produced by neoplasm cells or cells in their vicinity and can be quantified in body fluids and blood during cancer screening, diagnosis, and treatment monitoring. Antigens, cytoplasmic proteins, enzymes, hormones, receptors, oncogenes, and their derivatives could be considered biomarkers [16,17].

Ideal biomarkers possess certain characteristics such as high sensitivity and specificity to a specific tumor type, patient acceptance, positive and negative predictive values for predictive and prognostic benefits, and clinical validation through prospective trials. However, currently, there is no biomarker that fulfills all these ideal criteria. Biomarkers are categorized based on their application, including screening, detection of tumor presence or absence, prognosis, and identification of molecular targets for novel therapies [18,19]. 

The exploration for tumor biomarkers is enhanced by the analysis of body fluids such as saliva, urine, and blood/serum/plasma using minimally invasive and noninvasive methods. Currently, there is a particular emphasis on urine as a valuable waste material that is easily accessible, offers a larger volume, and possesses a lower proteome complexity compared to blood [20,21,22,23]. These urine-based biomarkers hold promising prospects for the detection and monitoring of OC, presenting opportunities for enhanced diagnostics and more effective management of the disease [17].

### 2.1. CA-125

CA125, initially reported in 1981, is a glycoprotein produced by the mucin 16 (MUC16) genes and can be identified using OC 125 monoclonal antibodies in cancerous ovarian tissues. The upper limit for CA125 is 35.0 U/mL in both premenopausal and postmenopausal patients [24].

#### 2.1.1. Role of CA-125 in Diagnosis and Prediction

The FDA guidelines endorse CA125 as a valuable protein biomarker for evaluating treatment response and monitoring ovarian cancer patients. CA125 levels correlate with clinical stage and survival outcomes, providing insights for clinical decision-making. However, CA125 alone does not accurately reflect tumor burden owing to potential secretion by non-tumor cells in an inflammatory environment [25].

Post-surgery, an elevated CA125 level (>35 U/mL) suggests residual disease, reduced chemotherapy sensitivity, and higher tumor malignancy. The Gynecologic Cancer Intergroup (GCIG) proposes criteria for assessing tumor remission and recurrence based on CA125 levels. A minimum 50% decrease sustained for four weeks classifies patients as responders, while complete responders have CA125 levels within the normal range (<35 U/mL). Ovarian cancer progression or recurrence is indicated by CA125 levels doubling with a one-week interval. Notably, persistent CA125 levels below 35 U/mL do not rule out residual disease and recurrence [26,27]. 

CA125 has emerged as a significant prognostic factor in the context of treatment outcomes following chemotherapy in women with advanced ovarian cancer. Post-initial cycle measurement and subsequent normalization of CA125 below 35 U/mL by the third cycle are crucial for prognosis. Lower CA125 levels and quicker normalization indicate a favorable chemotherapy response and extended progression-free survival. Decline in CA125 after neoadjuvant chemotherapy predicts positive debulking surgery outcome. Regular CA125 monitoring during first-line chemotherapy helps identify patients with reduced drug sensitivity, enabling timely treatment adjustments. CA125 accurately predicts disease progression following chemotherapy but does not impact survival afterward. Insulin signaling-induced CA125 oversecretion shows potential in predicting chemoresistance [28,29,30,31,32,33].

A recent study highlighted the importance of reducing nadir CA125 levels, as PFS was longer in patients with scores below 10 U/mL. The impact of maximal surgical effort on reduced scores remains uncertain [34,35].

#### 2.1.2. Limitations of CA-125 as a Diagnostic Marker

Relying solely on CA125 levels for epithelial ovarian cancer (EOC) diagnosis has limitations owing to false positives in healthy individuals and patients with benign conditions. Approximately 20% of EOC patients do not exhibit elevated CA125 levels, while lower CA125 levels are associated with earlier stages and improved outcomes. Circulating immune complexes (CICs) may contribute to lower CA125 concentrations by binding antibodies and inhibiting accurate detection. Considering these factors is crucial to avoid unnecessary burdens and to improve diagnostic accuracy in EOC patients [25,36,37,38,39].

Several studies, including the PLCO and UKCTOCS trials, have highlighted the limitations of CA125 as a screening tool for ovarian cancer. The PLCO trial demonstrated that combining CA125 screening with ultrasound did not significantly improve early detection or mortality outcomes compared to routine care. Moreover, false-positive results led to severe postoperative complications in 15% of patients [40,41]. Similarly, the UKCTOCS trial found no significant mortality benefit in the CA125 screening group compared to the control group [42,43].

### 2.2. HE4

HE4 is a glycoprotein produced by the WFDC2 gene and acts as a serine proteinase inhibitor. It serves as a potential biomarker for ovarian cancer (OC) and can be detected in the blood and urine of patients using enzyme immunoassay. HE4 exhibits overexpression in specific OC subtypes, with a 100% occurrence in endometroid tumors and 93% in serous OC. This characteristic enables its utility in distinguishing between various tumor types, aiding in the process of differential diagnosis. In 2008, the FDA authorized the use of HE4 for monitoring patients who have already been diagnosed with OC, while cautioning against its use in the screening of asymptomatic early-stage OC [17,44,45].

#### 2.2.1. Diagnostic Value of HE4

A recent study conducted at the University Hospital of Quebec City aimed to assess the performance of the preoperative plasma tumor markers, HE4 and CA125, in predicting cancer mortality in women with epithelial ovarian cancer (EOC). HE4 levels showed significant associations with important prognostic factors in both training and validation cohorts. HE4 demonstrated comparable performance to CA125 in predicting mortality in the training cohort, and a significant association was observed in the validation cohort. However, after adjusting for preoperative prognostic factors, the association became nonsignificant. Among women with serous ovarian cancer, HE4 showed a stronger association with mortality. HE4, along with other prognostic factors, may provide valuable information for predicting mortality in EOC, particularly in serous ovarian cancer cases [22].

A meta-analysis of 38 studies and 14,745 subjects evaluated serum HE4 as a diagnostic biomarker for ovarian cancer, showing promising discriminative power with acceptable sensitivity (0.79) and clinically meaningful specificity (0.92). Serum HE4 demonstrated an area under the curve (AUC) indicating its potential as a diagnostic tool. Additionally, post-test probabilities for serum HE4-positive and -negative subjects suggested its value in disease diagnosis [46].Another prospective study assessed HE4 alone and in combination with CA125 in 1229 symptomatic women, finding that the Risk of Ovarian Malignancy Algorithm (ROMA) had the best performance (AUC = 0.96). In women under 50, the combination of CA125 and HE4 showed superior sensitivity and specificity, while ROMA performed best in women over 50. HE4 alone had higher sensitivity but lower specificity than CA125 [47].

A study in 2018 investigated the prognostic significance of HE4 marker measurements during first-line chemotherapy in ovarian cancer patients. HE4 levels were found to predict platinum sensitivity and were associated with progression-free survival (PFS), overall survival (OS), and surgical outcome. HE4 demonstrated potential as a valuable biomarker for treatment response assessment and outcome prediction in ovarian cancer [48].

Another diagnostic study compared the accuracy of HE4, CA-125, Risk of Ovarian Malignancy Algorithm (ROMA), and Risk of Malignancy Index (RMI) in predicting ovarian cancer in patients with pelvic masses [49]. RMI takes the following factors into account: serum CA125 (CA125), menopausal status (M), and ultrasound score (U) [50] (see Table 1). ROMA had the highest overall accuracy, followed by HE4, CA-125, and RMI. HE4 and ROMA showed better identification of benign tumors compared to CA-125. In premenopausal women, HE4 and ROMA had higher specificity and negative predictive value, while in postmenopausal women, HE4 exhibited the highest specificity. HE4 and ROMA may serve as valuable diagnostic markers, particularly in specific patient populations and in differentiating benign from malignant masses [49].

A single-center study of 188 ovarian cancer patients found that higher levels of HE4 at diagnosis, after cytoreductive surgery, and during first-line chemotherapy were associated with increased risk of recurrence. Elevated HE4 levels were also observed in patients with larger residual tumors after primary surgery and in those who developed platinum resistance. Additionally, significantly higher HE4 levels were found in patients with neoplastic residues exceeding 10 mm at the diagnosis of a second recurrence [51].

Finally, a retrospective study of 89 EOC patients demonstrated that preoperative serum HE4 levels above 500 pM were significantly associated with lower 5-year overall survival (27% vs. 59%). These findings highlight the potential of HE4 as a prognostic marker for predicting ovarian cancer recurrence, treatment response, and survival outcomes [52].

#### 2.2.2. Complementary Role of HE4 to CA-125

Multiple studies have documented the efficacy of utilizing dual biomarkers to achieve high specificity and sensitivity in the combined analysis of both pre- and postmenopausal women with benign ovarian cysts.

Back in 2003, blinded tests were conducted on sera from 37 ovarian cancer patients (7 early stage and 30 late stage), 65 healthy asymptomatic controls, and 19 individuals with benign ovarian disease. The most common histology observed in the ovarian cancer patients was serous ovarian carcinoma (21 cases), and the most frequent stage was stage III (24 cases). Both CA125 and HE4 showed limitations as predictors of ovarian cancer, with HE4 failing to identify 7 cases and CA125 failing to identify 8 cases when using a 95% specificity threshold for positivity [53].

A total of 531 patients diagnosed with pelvic mass and scheduled for surgery were enrolled in a multicenter prospective study. Preoperative serum levels of HE4 and CA125 were measured to classify patients into low and high-risk groups for EOC. The study included patients with benign tumors, EOC, low malignant potential (LMP) tumors, non-EOC, and non-ovarian cancers. The model demonstrated high specificity and sensitivity in both the postmenopausal and premenopausal groups. It effectively categorized patients into high and low-risk groups, correctly classifying a significant proportion of EOC cases as high-risk [54].

A prospective study evaluating CA125 and HE4 measurements in blood and ascites found that although elevated levels were detected in baseline samples of patients with advanced high-grade serous EOC, these markers were unable to differentiate between patients with complete and incomplete resection or residual disease. Surgical treatment led to a decrease in tumor markers, likely influenced by ascites volume reduction and the long half-life of CA125. However, previous studies have shown that CA125 and HE4 assessments before and after chemotherapy initiation can predict treatment response and survival [55].

As demonstrated in a prospective multicentered trial by Moore et al., where the accuracy of RMI and ROMA was compared in diagnosing EOC, ROMA, utilizing HE4 and CA125, demonstrated higher sensitivity (94.3% at 75% specificity) than RMI (84.6%) in distinguishing between benign and EOC status. ROMA also showed superior sensitivity in stage I and II disease. These findings highlight the superiority of ROMA for identifying women with EOC [56].

CA125, HE4, and ROMA in a prospective study showed significant differences between benign and malignant cases, with elevated CA125 levels in endometriosis and ovarian fibromas/thecomas. HE4 levels varied between different types of cystadenomas/cystadenofibromas and endometriosis. ROMA was significantly elevated in certain benign masses compared to endometriosis. However, there were no significant differences in CA125, HE4, and ROMA levels between epithelial ovarian cancers (EOC) and metastatic tumors. Additionally, no significant differences were observed between different FIGO stages, except for the distinction between early (FIGO I-II) and advanced (FIGO III-IV) stages [57].

Monitoring EOC patients with serum HE4 levels showed similar performance parameters to CA125. The combination of HE4 and CA125 improved accuracy, sensitivity, and negative predictive value compared to using either marker alone. The study concluded that HE4 is equivalent to CA125 for monitoring EOC patients, and the combination of both markers provides superior monitoring capabilities [58]. HE4 levels were significantly elevated in ovarian and endometrial cancer patients compared to healthy controls, with the highest levels observed in serous carcinomas. Combining HE4 and CA125 provided the highest accuracy and sensitivity for distinguishing ovarian cancer patients from healthy controls and from those with ovarian endometriosis [59].

In a prospective study, HE4 demonstrated higher specificity for benign disease compared to CA125, and the combination of HE4 and CA125 showed the highest sensitivity for distinguishing invasive epithelial ovarian cancers from benign ovarian neoplasms [60]. HE4 was also identified as the top-performing individual biomarker for distinguishing between benign ovarian tumors and cancer, including borderline tumors, and a combined model including HE4, CA125, and age showed the highest performance [61].

The results suggest that HE4 is a potentially valuable biomarker for ovarian carcinoma, comparable to CA125, in distinguishing women with both localized and advanced ovarian cancer from healthy individuals. Additionally, the findings indicate that HE4 is superior to CA125 in differentiating patients with malignant ovarian disease from those with benign ovarian disease at high specificity.

### 2.3. CA 15-3

#### 2.3.1. Diagnostic Value of CA 15-3

In a 1988 study, elevated CA 15-3 levels (>30 U/mL) were found in 41% of cancer patients, particularly at advanced stages and in ovarian cancer cases, correlating with residual tumor, treatment response, and disease progression during chemotherapy [62].

#### 2.3.2. Complementary Role of CA 15-3 to CA-125

Significant differences were observed between the cancer group and both the benign and healthy control groups, indicating higher tumor marker levels in cancer patients. Combinations of tumor markers showed improved sensitivity compared to single markers, with the combination of CA72-4, CA15-3, and CA125 showing promise as a diagnostic tool for ovarian cancer [63]. In another study, an Artificial Neural Network (ANN) model was evaluated for the detection of early-stage ovarian cancer using multiple serum markers. The ANN model demonstrated improved performance in distinguishing early-stage cancer patients from healthy individuals, with the composite index derived from the ANN showing higher diagnostic power than CA125 alone. The combined use of multiple serum markers through the ANN model improved both sensitivity and specificity in detecting Stage I ovarian cancer, suggesting the potential of the ANN approach in enhancing early detection and diagnosis of ovarian cancer [64]. 

### 2.4. CA 19-9

CA19-9 is a sensitive marker for pancreatic, gastric, and hepatobiliary malignancies. Its potential use in OC screening was researched in recent studies. One of the six biomarkers Fahmy et al. studied, was CA19-9. They reported promising results with high sensitivity and specificity, thus revealing its potential for ruling out or ruling in the disease. In 120 patients with ovarian tumors and carcinoma, as well as 30 healthy controls, the levels of miRNA-204, CA125, CA19-9, hepcidin, microfibril-associated glycoprotein 2, and ferroportin were measured. MicroRNA-204, CA125, and CA19-9 showed increased levels in ovarian cancer patients, while hepcidin, microfibril-associated glycoprotein 2, and ferroportin levels were decreased. ROC analysis demonstrated that CA125 and CA19-9 exhibited high diagnostic performance individually, and the combination of microRNA-204, CA125, and CA19-9 had the highest diagnostic performance. Hepcidin, microfibril-associated glycoprotein 2, and ferroportin had weaker diagnostic performance [65].

Another retrospective study analyzed data from 314 patients diagnosed with mucinous ovarian tumors. Preoperative serum levels of CA19-9, CA-125, and CEA were assessed, and their diagnostic performance was evaluated using receiver operating characteristic curves. Associations between clinicopathological factors and biomarker levels were also investigated. The results showed that elevated CA19-9, CA-125, and CEA levels, along with tumor size, influenced tumor pathology. The mucinous ovarian tumors with larger sizes and elevated biomarker levels demonstrated a positive correlation with increased risk. Among the three markers, CA-125 provided the highest diagnostic performance in differentiating between benign, borderline, and malignant mucinous ovarian tumors. Preoperative elevation of CA19-9, CA-125, and CEA, along with tumor size, can serve as useful predictors in distinguishing tumor types [66]. 

### 2.5. hCG

Human chorionic gonadotropin (hCG) is expressed in various tumor types, including OC, making it a potential prognostic and therapeutic target. In biological fluids, hCG exists in different isoforms with varying biological activities, including intact hCG, cleaved hCGn, free β subunits (hCGβ), inactive hCGα, β-core fragment, and nicked free β-subunit (hCGβn) [67]. 

#### 2.5.1. Prognostic Significance of hCG

The expression of human chorionic gonadotropin (hCG) in epithelial ovarian cancer (EOC) has been investigated in two studies. One study found significantly elevated levels of hCG mRNA and protein expression in EOC cases, with higher expression in advanced-stage EOC samples. Increased hCG expression and tumor metastasis were identified as independent unfavorable prognostic factors for overall survival [68]. Another study examined serum hCG levels in patients with ovarian tumors and found that 68% of ovarian cancer tissues were hCG-positive, with variations among histological subtypes. Tumor grade and stage significantly influenced hCG expression. Patients with hCG(+) and LH-R(+)/FSH-R(−) tumors exhibited better 5-year survival rates [69]. Additionally, LH/hCG receptor mRNA and protein expression were investigated in various ovarian tumors, revealing positive expression in a substantial proportion of ovarian cancers, borderline tumors, and benign cystadenomas. LH/hCG receptor-positive tumors were associated with a more favorable prognosis, particularly in well-differentiated cancer phenotypes. These findings suggest the potential of hCG and LH/hCG receptor as targets for innovative cancer treatments, enhancing effectiveness while minimizing adverse effects [70].

#### 2.5.2. hCG as a Potential Diagnostic Marker

The expression and diagnostic value of human chorionic gonadotropin (hCG) and its subunit β-hCG have been investigated in ovarian cancer patients. Vartiainen et al. found elevated levels of hCGβ in 29% of ovarian cancer patients, with increasing frequency in advanced stages and specific cancer types. CA125 levels were elevated in 79% of patients and correlated with cancer stage. Both hCGβ and CA125 showed strong associations with prognosis, but in a multivariate model, only hCGβ, stage, and grade remained significant. A cutoff level of 2 pmol/L for hCGβ distinguished patients with different prognoses, particularly in advanced-stage disease [71]. Another study monitored β-hCG levels in ovarian cancer patients before and after surgery, revealing significant differences between cancer stages. However, the diagnostic value of β-hCG was deemed unreliable, with false positive and false negative results [72]. Immunohistochemical analysis demonstrated increased β-hCG expression in metastatic ovarian carcinoma tissues and its association with unfavorable clinical features, including advanced stage, larger tumor size, poor differentiation, and high-grade serous carcinoma. These findings suggest that β-hCG expression is linked to aggressive clinical features in ovarian cancer [73].

### 2.6. Inhibin

Inhibins, consisting of α and β subunits, are growth factors involved in fertility regulation primarily produced by ovarian follicles. Total inhibin measurement is crucial in investigating ovarian cancer as different subtypes produce varying amounts of inhibin species. Elevated total inhibin levels are observed in postmenopausal women with granulosa cell tumors and mucinous epithelial cancers. In combination with CA125, inhibin improves ovarian cancer detection, particularly for specific subtypes. However, inhibin has limited effectiveness as a marker in premenopausal women [74]. A study evaluated serum inhibin concentrations in postmenopausal women with ovarian cancer using the αC inhibin immunofluorometric assay (IFMA) and CA125, demonstrating higher sensitivity and comparable or better specificity than previous methods [75]. Inhibin A and B levels are typically undetectable in postmenopausal women without ovarian malignancy, while combining inhibin B and anti-Mullerian hormone (AMH) shows promise for diagnosing and monitoring granulosa cell tumors. Total inhibin, including free alpha subunit and inhibin A and B, may be useful for some serous and mucinous epithelial carcinomas when combined with CA-125. However, the utility of inhibin measurement for premenopausal women and early-stage tumor detection remains uncertain [76].

Inhibin is a valuable tumor marker in ovarian granulosa cell tumors (GCTs), with elevated serum levels observed in GCT patients. Assays such as inhibin RIA and inhibin ELISA have been developed, with the latter showing potential for widespread use. Total inhibin levels are typically low in healthy postmenopausal women but can distinguish ovarian cancer cases. Combining inhibin with CA125 improves detection, achieving high sensitivity and specificity in identifying ovarian cancers [77]. In normal postmenopausal women, inhibin levels are often undetectable, but detectable levels show a dose-response relationship with inhibin A. In early-stage mucinous carcinomas, inhibin levels have been detected, suggesting potential sensitivity in early-stage disease. The underlying biological and molecular mechanisms behind elevated inhibin levels in ovarian cancer are not fully understood, but elevated gonadotropins are believed to play a role [78]. GCTs have a propensity for metastasis and recurrence, and monitoring inhibin B, a biomarker reflecting tumor burden, can be useful in assessing treatment response and disease recurrence [79]. Inhibin, particularly inhibin B, is a valuable circulating tumor marker for GCTs, and further research is needed to explore the molecular pathogenesis of ovarian tumors and the significance of inhibin in their development [80].

### 2.7. AFP

Alpha-fetoprotein (AFP) is a fetal serum protein that can serve as a marker for detecting cancerous growths. However, elevated AFP in epithelial ovarian carcinoma (EOC) can lead to misdiagnosis, particularly in young women, as high AFP levels are uncommon in EOC. This poses challenges for accurate diagnosis and emphasizes the need for careful evaluation. A study investigated AFP-producing EOC and found it to be associated with aggressive behavior and poor prognosis. AFP expression was confirmed in all cases, suggesting differentiation into yolk sac components. Serum AFP levels are not routinely examined in older women, potentially leading to missed diagnoses [81]. Another study evaluated multiple tumor markers, including AFP, and found that they effectively distinguished ovarian cancer from benign cases and healthy individuals [82]. AFP-producing ovarian tumors are rare and present diagnostic difficulties. The rarity and poor prognosis of AFP-producing tumors highlight the need for improved management strategies [83]. Alpha-fetoprotein, along with other serum markers, is commonly used for ovarian germ cell tumors (OGCT) but has limitations in early-stage screening. Monitoring AFP and βhCG levels is important for prognosis, and inhibin B is valuable for monitoring granulosa-theca cell tumors [84]. A study demonstrated the high diagnostic accuracy of a combined approach involving transvaginal sonography, color Doppler, and tumor marker tests for ovarian cancer diagnosis [85]. 

### 2.8. LDH

Lactate dehydrogenase (LDH) is an enzyme involved in glycolysis that converts pyruvate to lactic acid. Studies have shown higher LDH blood levels in the blood of ovarian cancer patients, indicating its release by neoplastic cells into the surrounding medium.

Serum LDH levels were found to be significantly higher in OC patients in a prospective study. Specifically, a sensitivity of 60%, specificity of 86%, positive predictive value of 70%, and negative predictive value of 75% were observed using as cutoff level the 450 IU/mL for serum LDH. Authors suggest that serum LDH levels could serve as a reliable biochemical marker for differentiating ovarian cancer from benign tumors [86]. 

In 2017 Bastani et al. assessed the diagnostic value of serum markers (prostasin, CA125, LDH, AFP, hCG + β) in epithelial ovarian cancer (EOC) and their potential for distinguishing EOC from benign tumors and healthy individuals. The findings demonstrated that serum levels of prostasin, LDH, and CA125 were significantly higher in EOC patients compared to those with benign tumors and healthy controls. LDH levels increased with higher stages of EOC. Combining prostasin and LDH with CA125 improved the prediction of EOC status. The multi-marker approach showed promise for more accurate differential diagnosis in EOC patients [87].

## 3. Emerging Tumor Markers

### 3.1. MicroRNAs

**Figure 2:**
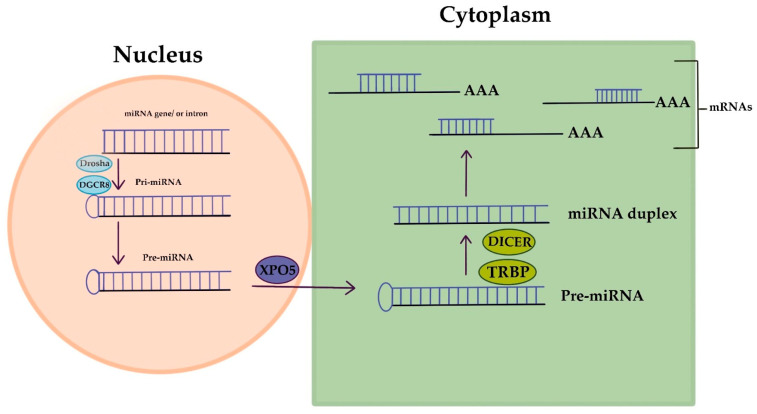
MicroRNA’s creation and way of action.

MicroRNAs (miRNAs) are short RNA molecules that regulate gene expression and play crucial roles in various biological processes. The production and way of action of miRNA is displayed in Figure 2. MiRNA genes are transcribed to produce pri-miRNA, which is cleaved to form pre-miRNA. In the cytoplasm, pre-miRNA is further cleaved to generate a miRNA duplex. The mature miRNA regulates gene expression by targeting mRNA for cleavage or translation repression based on miRNA–mRNA complementarity. Their dysregulation is associated with numerous human conditions, including cancer [88]. Aberrant miRNA expression in ovarian cancer has diagnostic and prognostic potential as circulating miRNAs (cirMiRs), offering non-invasive biomarkers [89]. MiRNAs regulate gene expression by targeting multiple genes, making them valuable for understanding gene behavior [90,91]. MiRNAs are stable in the circulation, bound tochaperone protein Argonaute 2(Ago2) or enclosed in extracellular vesicles, resisting degradation by ribonucleases [92,93]. Over 2500 miRNAs have been identified, capable of targeting multiple genes within pathways, providing valuable insights into gene behavior [90,91,94]. Let-7 and miR-200 miRNA families are implicated in OC development. Let-7 has potential for selecting chemotherapy, while miR-200’s role in chemo-sensitivity is uncertain. miRNAs hold promise as chemotherapy response predictors, but further validation is needed. Plasma/serum miRNAs offer potential for early OC diagnosis, but before clinical utility additional research is required [94]. Dysregulated miRNAs in OC act as tumor suppressors or oncogenes. Low expression of miR-processing enzymes is linked to advanced tumor stage and poor outcomes. Let-7 and miR-200 families are frequently altered in OC. Various miRNAs have diagnostic and therapeutic potential. Serum miRNA panels show promise for OC diagnosis and monitoring. miRNAs are valuable tools for OC management [10]. Aberrant miRNA expression in OC is associated with chemoresistance, including let-7e, miR-30c, miR-125b, miR-130a, miR-335, miR-340, miR-381, and miR-520f [95].

MiRNA expression profiles show diagnostic potential in ovarian cancer, with specific miRNAs differentially expressed in OC samples. Circulating miRNAs in blood and urine are promising diagnostic markers. MiRNAs also correlate with histotypes, chemoresistance, and prognosis, offering insights into disease progression and chemotherapy outcomes in OC [96]. Altered miRNA expression in OC correlates with disease stage, treatment response, and overall survival. miR-21, miR-200a, and miR-200c have diagnostic and prognostic value, while let-7f and miR-141 are associated with worse progression-free survival. miR-193a acts as a tumor suppressor [89]. In an enlightening study by Yokoi et al., the discrimination of early-stage ovarian cancers from benign tumors was achieved with an impressive sensitivity of 86% and specificity of 83% by employing a panel of eight miRNAs. Furthermore, the presence of miRNAs was detected in EVs isolated from cultured ovarian cancer cell lines [97].

### 3.2. DNA Methylation Patterns

DNA methylation markers offer early detection potential in OC, unlike CA125 [98,99]. The utilization of cell-free DNA (cfDNA) methylation markers shows promise in identifying early-stage OC patients within the average-risk population. Since early-stage OC patients typically remain asymptomatic, incidental diagnosis is common. Consequently, the development of effective DNA methylation markers holds promise for early OC detection and requires continued investigation to enhance clinical applicability [100,101]. Frequent genetic alterations in cancer involve hypermethylation of tumor suppressor promoters and hypomethylation of oncogenes. Methylation-specific PCR (MSP) stands as a highly sensitive technique, capable of detecting one methylated allele among 1000 unmethylated alleles. The occurrence of promoter hypermethylation escalates as the disease progresses [102,103].

Multiplexed methylation-specific PCR (MSP) of cfDNA for seven genes showed high sensitivity (85%) and specificity (91%) for early-stage ovarian cancer compared to CA125 alone [104]. Widshwendter et al. developed a three-DNA-methylation-serum-marker panel using targeted ultra-high coverage bisul-fite sequencing. The panel successfully differentiated high-grade serous ovarian cancer patients from healthy women or those with benign pelvic masses, achieving a sensitivity of 41.4% and specificity of 90.7%. When applied to serum samples collected 1–2 years before ovarian cancer diagnosis, the methylation panel showed a sensitivity of 16.7% and specificity of 96.9% [98].

DNA methylation is a useful marker for cancer cell fraction analysis, providing advantages in terms of time, cost, and independence from allelic status. This approach, along with other markers, reduces reliance on pathologists and enables efficient analysis of ovarian cancer cell fractions [105]. A study identified DNA methylation markers (COL23A1, C2CD4D, and WNT6) with high sensitivity and specificity for early ovarian cancer (OC) detection. The markers exhibited aberrant methylation patterns in early-stage OC and showed promise in discriminating OC from healthy individuals. The panel demonstrated potential as a complementary approach for early OC diagnosis, particularly in CA125-negative samples [98]. Late-stage methylation markers show limited utility in early-stage ovarian cancer (OC) detection, while early-stage markers demonstrate satisfactory discrimination. Hypomethylated regions display reversal to baseline levels in late-stage OC. Early-stage methylation markers remain stable during cancer progression, offering potential for OC detection across all stages [106]. SIM1 and ZNF154 genes were identified as potential methylation markers for ovarian cancer cell fraction estimation. ZNF154 was validated as a reliable marker, offering a cost-effective and efficient method for assessing ovarian cancer cell fraction using pyrosequencing [107].

Ovarian clear cell carcinoma (OCCC) can be classified into two distinct clusters based on DNA methylation patterns. Cluster 1 is associated with advanced stage, poorer outcomes, TP53 mutation, and macroscopic residual disease, while Cluster 2 is characterized by early stage, aneuploidy, ARID1A/PIK3CA mutation, and longer overall survival. Immune-related pathways and ARID1A mutations contribute to the molecular and clinical heterogeneity of OCCC [108]. Ovarian cancer DNA methylation analysis identified 250 prognosis-related loci, revealing six subtypes with distinct patterns and prognoses. Subtype 2 had the highest methylation and best prognosis, while subtypes 4 and 5 had lower methylation and poor prognoses. Hypomethylation correlated with worse outcomes. These subtypes could serve as biomarkers for personalized treatment and prognosis prediction [109]. A study identified 89 CpG sites associated with epithelial ovarian cancer (EOC) risk, including 12 CpG sites and five genes (MAPT, HOXB3, ABHD8, ARHGAP27, and SKAP1) showing consistent associations. Methylation at these sites may regulate gene expression and influence EOC risk, particularly for serous and high-grade serous ovarian cancer. Integration of genetic, methylation, and gene expression data provides insights into EOC development and potential personalized treatment targets [110]. HOXA10 and HOXA11 genes show significant DNA methylation differences in ovarian cancer, with HOXA11 methylation associated with poor prognosis and residual tumor. HOXA10 methylation is higher in poorly differentiated cancers. Low HOXA11 methylation correlates with minimal residual tumor and serves as an independent prognostic marker. Methylation frequency increases from non-neoplastic to primary ovarian cancer, highlighting their diagnostic and prognostic potential [111].

Comprehensive analysis revealed (hypomethylated-upregulated) HOUP genes associated with ovarian cancer progression and potential prognostic markers, while (hypermethylated-downregulated) HEDW genes were enriched in cancer-related pathways. Dysregulated hub genes and negative correlations with methylation levels were identified, providing insights into ovarian cancer epigenetic alterations and biomarkers [112]. Cervical scrapings from Pap tests showed significant hypermethylation in five genes in OC patients. An integrated model incorporating methylation levels predicted OC risk with high sensitivity and specificity, offering potential for enhanced detection of female genital tract malignancies [113]. cfDNA methylation analysis identified specific differentially methylated regions (DMRs) associated with OC. A customized methylation panel revealed OC-specific DMRs with distinct methylation patterns, suggesting their potential as diagnostic and prognostic markers [114]. Finally, in a comprehensive analysis a validated serum marker panel using targeted bisulfite sequencing showed high sensitivity and specificity, suggesting the potential of DNA methylation patterns for early OC detection [98].

### 3.3. Circulating Tumor Cells

Circulating tumor DNA (ctDNA) allows non-invasive detection of ovarian cancer mutations, such as PIK3CA and KRAS, with potential as diagnostic and prognostic markers in liquid biopsy. Differentiated from lymphocyte DNA, cfDNA exhibits characteristic fragmented size [115]. Research efforts have primarily focused on analyzing the fraction of cfDNA originating from tumors, known as circulating tumor DNA (ctDNA) [116]. ctDNA is primarily released from tumor cells through apoptosis [117,118]. Recent advancements in deep sequencing and droplet digital PCR (ddPCR) techniques have enabled the detection of specific mutations, loss of heterozygosity (LOH), DNA hypermethylation, copy number variations, and even the presence of single nucleotide variants in minute quantities of ctDNA [119,120,121,122,123].

Swisher et al. detected tumor-specific TP53 mutations in cfDNA using traditional PCR, with a 30% detection rate in plasma or serum samples. ctDNA analysis shows potential as a non-invasive method for identifying cancer-specific mutations across different stages [124]. Advanced sequencing technologies, such as tagged amplicon sequencing (TAm-Seq) and duplex sequencing, enhance ctDNA detection with high sensitivity (as low as 2% allelic fractions) and specificity (97% for TAm-Seq). However, the applicability in early-stage cancers and the balance between sensitivity and specificity require further investigation. Duplex sequencing reveals low-level mutant TP53 events in peritoneal fluid, suggesting normal physiological processes involve mutant TP53 [125,126,127,128]. The integration of ctDNA with CA125 in a multi-cancer investigation achieved a high sensitivity of 98% for detecting ovarian cancer, primarily in advanced-stage tumors [129]. PIK3CA and KRAS mutations in ctDNA serve as prognostic markers and indicate outcomes in epithelial ovarian cancer patients. ctDNA detection rates correlate with advanced stage and peritoneal cytology, while ctDNA presence at primary treatment predicts shorter recurrence-free survival [130].

ctDNA detection in EOC patients correlates with advanced stages, high-grade disease, disease progression, and higher mortality rates. CtDNA outperforms CA-125 as a prognostic indicator for recurrence, with its presence after surgery strongly associated with reduced recurrence-free survival. CtDNA provides valuable insights for risk assessment and monitoring of EOC recurrence. Genomic profiling reveals frequent mutations in TP53, ARID1A, KRAS, and PIK3CA [131]. Finally, a systematic review of eight studies involving 627 ovarian epithelial cancer patients found that ctDNA is significantly associated with decreased overall survival (OS) and progression-free survival (PFS). Serum-derived ctDNA showed a strong relationship with reduced OS, while plasma-derived ctDNA had some heterogeneity. The analysis also indicated that ctDNA could serve as an independent risk factor and a potential biomarker for evaluating ovarian cancer prognosis [132].

## 4. Conclusions

Tumor markers, particularly CA-125, are widely used in the diagnosis of ovarian cancer but have limitations in sensitivity and specificity, especially in early-stage and certain subtypes of the disease. Additional biomarkers such as HE4, microRNAs, DNA methylation patterns, and circulating tumor cells show promise in improving both diagnostic accuracy and early detection. Combining multiple markers, like CA-125 and HE4, enhances diagnostic accuracy and risk stratification. Challenges in clinical implementation include standardization, validation, and algorithm development. Personalized medicine guided by biomarker profiles offers tailored treatment and improved outcomes. Ongoing research is needed to address limitations and develop novel markers. Integration of multiple markers and personalized medicine approaches will lead to more accurate diagnosis, better risk stratification, and improved outcomes for ovarian cancer patients.

## Figures and Tables

**Table 1:** Risk of malignancy index (RMI) scoring system [50].

Parameter	RMI 1	RMI 2	RMI 3	RMI 4
**Ultrasonography score (U)**				
No feature	0	1	1	1
1 feature	1	1	1	1
≥2 features	3	4	3	4
**Menopausal status (M)**				
Premenopausal	1	1	1	1
Postmenopausal	3	4	3	4
**CA-125 (U/mL)**	Absolute value in U/mL			
**Tumour size (S)**				
<7 cm	-	-	-	1
≥7 cm	-	-	-	2

Formula for RMI 1, 2 and 3: U × M × CA-125. Formula for RMI 4: U × M × CA-125 × S.

## Data Availability

Data used in this study are presented within the manuscript.
